# Low Natural Parasitism of the Invasive *Halyomorpha halys* Versus Strong Native Suppression of *Palomena prasina*: Evidence from a Three-Year Survey in Northwestern Türkiye

**DOI:** 10.3390/insects16121212

**Published:** 2025-11-28

**Authors:** I. Oguz Ozdemir, Muhammed Fatih Şılbır, Eren Karadağ, Abdullatif Alaybay, Göksel Özer, Francesco Tortorici, Vaughn M. Walton, Furkan Dogan

**Affiliations:** 1 Department of Plant Protection, Faculty of Agriculture, Sakarya University of Applied Sciences, 54580 Sakarya, Türkiye; 2 Department of Horticulture, Oregon State University, 4017 Agriculture and Life Sciences Building, Corvallis, OR 97331, USA; vaughn.walton@oregonstate.edu; 3 Department of Plant Protection, Faculty of Agriculture, Bolu Abant Izzet Baysal University, 14020 Bolu, Türkiye; gokozer@gmail.com; 4 Department of Agricultural, Forest and Food Sciences, University of Torino, Largo Paolo Braccini 2, 10095 Grugliasco, Italy; francesco.tortorici@unito.it

**Keywords:** hazelnut, Hemiptera, Pentatomidae, green shield bug, brown marmorated stink bug, biological control, natural enemies, egg parasitoid, parasitism rate, discovery efficiencies, exploitation efficiencies

## Abstract

This study assessed the parasitism rates and species composition of egg parasitoids associated with *Palomena prasina* and *Halyomorpha halys* in Northwestern Türkiye, one of the country’s main hazelnut production areas. Between 2022 and 2024, a total of 15,051 *P. prasina* eggs and 8490 *H. halys* eggs were collected from 65 hazelnut orchards and neighboring fruit orchards in Northwestern Türkiye. Egg masses were maintained under laboratory conditions, and emerging parasitoids were identified using morphological and molecular methods. The total parasitism rate on *P. prasina* was 17.39%, primarily caused by *Trissolcus belenus* (42.51%), *Telenomus turesis* (26.47%), *Trissolcus cultratus* (23.95%), *Telenomus truncatus* (6.80%), and *Anastatus bifasciatus* (0.27%). In *H. halys,* the total parasitism rate was 1.98%, and all parasitoids that emerged were identified as *T. belenus* (26.19%), *T. truncatus* (16.67%), and *A. bifasciatus* (15.48%). The highest discovery (24.88%) and exploitation efficiency (89.83%) for *P. prasina* were observed for *T. belenus*. Although native parasitoids are generally considered ineffective against *H. halys* due to evolutionary mismatch, their natural parasitism on *P. prasina* eggs is promising for long-term management. The findings clarify parasitoid composition and natural parasitism patterns, demonstrating that conserving and augmenting native parasitoids can contribute to more sustainable management and reduced insecticide dependence in Turkish hazelnut orchards.

## Simple Summary

The native green shield bug (*Palomena prasina*) and the invasive brown marmorated stink bug [*Halyomorpha halys* (Hemiptera, Heteroptera, Pentatomidae)] are important stink bug species causing serious yield and quality losses in Turkish hazelnut orchards. Although intensive insecticide applications are currently used to control both pests, these chemicals harm beneficial arthropods and pose risks to human health and the environment. Biological control, a promising alternative, particularly through egg parasitoids, represents a sustainable management approach against *P. prasina* and *H. halys* populations. Between 2022 and 2024, a total of 23,541 eggs were collected from hazelnut and neighboring fruit orchards in northwestern Türkiye to determine which parasitoid species parasitized these eggs and how effective they were. Five parasitoid species were identified, and the parasitism rate was found to be much higher in *P. prasina* (17.39%) than in the invasive *H. halys* (1.98%). The most common parasitoid species, *Trissolcus belenus*, showed the highest discovery and exploitation efficiency. These findings indicate that native parasitoids provide natural control of *P. prasina*, whereas their impact on *H. halys* remains limited. Determining which parasitoids are active in hazelnut orchards will support long-term hazelnut quality and yield in Türkiye.

## 1. Introduction

Stink bugs (Hemiptera: Pentatomidae) are economically important pests of field crops, vegetables, and fruit trees, whose feeding on plant tissues causes both direct damage and facilitates pathogen entry, leading to significant yield and quality losses [1,2,3,4]. In Turkish hazelnut orchards, more than 15 true bug species (Hemiptera: Acanthosomatidae, Coreidae, and Pentatomidae) have been reported [5,6,7]. Among them, the green shield bug (GSB), *Palomena prasina* L. (Hemiptera: Pentatomidae), is the most dominant species due to its widespread occurrence and high population density, accounting for over 85% of the stink bug community in hazelnut orchards in Türkiye, and its population levels generally exceed the economic threshold [5,8]. Although GSB is a polyphagous species, it is known to cause substantial yield and quality losses in hazelnuts not only in Türkiye [5,8,9,10], but also in other hazelnut-producing countries [11,12,13,14,15]. On the other hand, the brown marmorated stink bug (BMSB), *Halyomorpha halys* (Stål) (Hemiptera: Pentatomidae), has rapidly expanded its distribution across multiple continents, including North America [16,17,18], Europe [19,20], and North Africa [21]. With more than 300 host plants, including fruits, nuts, field crops, and vegetables, BMSB has caused serious global economic losses [22,23]. In Türkiye, BMSB was first detected in 2017 in Istanbul [24] and Artvin province, a hazelnut-producing region bordering Georgia [25]. Since then, the species has spread along the Black Sea region, Türkiye’s main hazelnut production area, leading to sudden outbreaks [26,27]. Molecular studies revealed that Turkish populations originated from a single mitochondrial haplotype but show high genetic diversity based on SCoT markers [28]. Similar to Türkiye, other major hazelnut producers, including the United States of America [29], Italy [11], and Georgia [30], have also suffered severe BMSB damage, making it a global threat to the sustainability of hazelnut yield and quality [31].

Both GSB and BMSB can complete their life cycle on hazelnut; while GSB is univoltine, BMSB may produce a partial second generation depending on photoperiod and temperature in Türkiye [26,29,32]. Feeding before kernel expansion causes abortion and blank nuts; feeding during expansion produces shriveled or malformed kernels, and feeding on mature nuts leads to corking damage [29,32]. Corked kernels, detected after shelling, reduce nut quality and export value [5,31,33]. Historical surveys conducted between 1996 and 2000 reported up to 20% corking in Türkiye [5], with 7.44% average kernel damage in 2014–2016 [33]. In Italy and Georgia, GSB damage ranged from 1.3 to 4.0% [11,12,34]. In contrast, BMSB damage reached 74.82% compared to 39.49% by GSB [11]. Currently, in some Turkish hazelnut orchards with BMSB outbreaks, corked damage has reached up to 70%, whereas in Oregon (USA) orchards with high BMSB populations, this damage is around 50% [35]. Considering that Türkiye accounts for ~60% of global hazelnut production [36,37], these threats underscore the urgency of reassessing control strategies. This is due to the actual and potential yield and quality losses posed by both stink bug species, particularly under the threat of increasing BMSB populations [6,31].

Currently, management of GSB and BMSB relies mainly on intensive insecticide use, with two–three applications per season for GSB and four–five applications for BMSB in Turkish hazelnut orchards [10,38,39]. However, broad-spectrum insecticides harm beneficial arthropods, raising environmental and health concerns [7,40,41]. Biotechnical methods have been tested to reduce chemical use [42,43], yet further research is needed, particularly on attract-and-kill strategies. In this context, biological control, especially of egg parasitoids, represents a sustainable alternative for managing GSB and BMSB populations [7,40,44].

Egg parasitoids (Hymenoptera) are considered the most effective and widespread natural enemies of hemipteran pests, as they attack their hosts during their most vulnerable biological stage, before the insects emerge [45,46]. In this context, egg parasitoids belonging particularly to the families Scelionidae, Encyrtidae, and Eupelmidae stand out, and species from these families have been shown to successfully parasitize the eggs of GSB and BMSB [40,47,48,49,50,51,52]. In Türkiye, studies on the egg parasitoids of GSB are limited. In addition to *Trissolcus grandis* (Thomson), a junior synonym of *T. belenus* (Walker) (Hym.: Scelionidae) sensu [53], reported as an egg parasitoid of GSB [54], *T. cultratus* (Mayr), *T. belenus* (Walker), *Trissolcus* sp1, *Telenomus turesis* Walker, and *T. truncatus* (Nees von Esenbeck) (formerly referred to as *Telenomus* sp1) (Hym.: Scelionidae) were identified parasitizing sentinel eggs of GSB in the main hazelnut production areas of Türkiye, with *T. cultratus* being determined as the overall predominant species [7,55]. In Switzerland, *T. semistriatus* and *T. turesis* (senior synonym of *T. chloropus*) have been found in sentinel eggs of GSB [47], while in Northwestern Italy, including hazelnut orchards, naturally laid GSB eggs were parasitized by *T. kozlovi*, *Anastatus bifasciatus* (Geoffroy) (Hym.: Eupelmidae), *T. truncatus*, *T. belenus*, *T. cultratus*, *T. japonicus*, and *T. mitsukurii* [40,50,51]. Similarly, in Türkiye, native species parasitizing BMSB eggs have been reported as *T. turesis* [56] and *A. bifasciatus* [57]. Among the egg parasitoids found in BMSB’s native range, *T. japonicus* (in China and Japan) and *T. mitsukurii* (in Japan) are particularly notable [58]. While populations of this invasive species are partially suppressed by natural enemies in their native range, the pressure from natural enemies disappears outside these areas, and invasions largely occur due to this reason [48,59,60]. Therefore, in invaded areas, native natural enemies that have not co-evolved with the pest are present instead of co-evolved natural enemies [61,62,63]. Recent studies indicate that host–parasitoid interactions are strongly influenced by co-evolutionary history and geographic context. Co-evolved parasitoids exploit host cues more efficiently, whereas non-co-evolved populations show reduced parasitism in novel environments [64]. For instance, comparative studies on *T. japonicus* and *T. cultratus* further reveal that evolutionary history and spatial isolation shape developmental success and competitive strategies [65]. In regions invaded by BMSB, only a few native parasitoids (*Anastatus* spp., *Telenomus* spp., *Trissolcus* spp., *Gryon* spp., and *Ooencyrtus* spp.) have been reported in the literature as successfully developing in BMSB eggs [47,48,66,67,68,69]. Indeed, despite its presence in Switzerland for over a decade, only two native parasitoid species (*T. cultratus* and *A. bifasciatus*) have been identified [47]. However, parasitism rates caused by these native species have generally been low [49]. Nevertheless, despite BMSB’s relatively recent establishment in North America, some native parasitoids (e.g., *Telenomus* spp.) have shown partial developmental success in fresh eggs, which may be associated with the beginning of adaptation to the host [70,71,72,73].

Although parasitism rates of native GSB in Türkiye’s main hazelnut regions have been determined using frozen sentinel egg masses [7], the lack of host-related chemical cues in these eggs may reduce parasitoid detection, leading to underestimation of actual rates and species diversity [45,70,71,74]. Thus, naturally laid eggs provide a more accurate reflection of ecological interactions and species behavior [45]. Given the recent release of *T. japonicus* against BMSB in Türkiye as part of classical biological control, assessing its effectiveness alongside native parasitoids in hazelnut orchards is crucial [75]. As Türkiye is the world leader in hazelnut production, and spraying remains the only short-term option for controlling GSB and BMSB, the development of environmentally friendly and sustainable control methods is of great importance. Therefore, this study aims to collect naturally laid GSB and BMSB egg masses from hazelnut orchards and neighboring fruit orchards in the Northwestern Black Sea region to determine natural parasitism rates and the composition of parasitoid species.

## 2. Materials and Methods

### 2.1. Collection of Naturally Laid Egg Masses of GSB and BMSB

In order to assess the parasitism of GSB and BMSB eggs, a three-year survey was conducted in Sakarya (Karasu, Kocaali, Geyve, and Arifiye districts) and Düzce (Akçakoca, Gümüşova, and Cumayeri districts), which are among the main hazelnut production areas of Türkiye. These studies were carried out in Sakarya and Düzce in 2022 and 2023, and only in Sakarya in 2024. Egg masses of GSB and BMSB were collected from a total of 65 hazelnut orchards between May and July. Surveys were conducted at regular weekly intervals during each sampling period. During field studies, the canopy parts of each hazelnut tree (*Corylus avellana* L.) were carefully examined at a height of 1–2 m [40]. In addition, egg masses were also collected from different fruit and forest tree species in nearby orchards or surrounding areas, including blackthorn (*Prunus spinosa* L.), pear (*Pyrus communis* L.), walnut (*Juglans regia* L.), hornbeam (*Carpinus betulus* L.), kiwifruit (*Actinidia deliciosa* Planch), and cherry (*Prunus avium* L.). The collected egg masses were placed in labeled plastic containers (10 × 10 × 5 cm) and taken to the Entomology Laboratory of the Faculty of Agriculture, Sakarya University of Applied Sciences.

### 2.2. Rearing and Observation of GSB and BMSB Egg Masses in the Laboratory

After being transported to the laboratory, the egg masses collected from the field were kept for six weeks in a climate-controlled chamber set at 25 ± 1 °C, 70% relative humidity, and a 16:8 h light–dark photoperiod [7]. During this period, the egg masses were checked every two days to regularly monitor the emergence of either parasitoids or GSB and BMSB nymphs. All egg masses were examined under a ZEISS Stemi 305 (Carl Zeiss Microscopy GmbH, Jena, Germany) stereo microscope at 50× magnification, and each egg was classified into one of five categories based on the study of Moraglio et al. [40]: (1) Hatched eggs were defined as empty eggs showing at least one characteristic morphological sign of emergence, such as an opening in the cap, structural folding, or a slit, indicating that the individual had successfully emerged [76,77]. (2) Parasitized eggs were characterized by internal discoloration due to parasitoid development and the presence of an irregular exit hole [77]. (3) Predated eggs were those completely emptied and bearing one or more needle-like punctures (stylet marks), suggesting consumption by predators with piercing–sucking mouthparts. (4) Broken eggs were defined as eggs with physical breakage or damage on the outer surface (chorion) and no internal contents. (5) Unhatched eggs were intact eggs from which no emergence was observed and for which the cause of mortality could not be morphologically determined [40].

### 2.3. Determination of Egg Parasitoid Performance

Parasitoid performance was evaluated using three indices: parasitism rate, discovery efficiency, and exploitation efficiency. The parasitism rate for each egg parasitoid species was calculated as the ratio of the number of parasitized eggs to the total number of eggs. Discovery efficiency was calculated as the ratio of the number of egg masses discovered by the parasitoid (i.e., those containing at least one parasitized egg) to the total number of collected egg masses. Exploitation efficiency was defined as the ratio of parasitized eggs to the total number of eggs in only those egg masses that were found to be parasitized [78]. Parasitism rate, discovery efficiency, and exploitation efficiency were analyzed using generalized linear models (GLMs) with a binomial distribution and logit link in IBM SPSS (Version 27.0.1) Statistics. Because GSB and BMSB represent distinct host species with different sampling periods and egg mass numbers, these datasets were analyzed separately. For each year, data from all provinces and districts were evaluated together, and means were separated at *p* < 0.05 using the Bonferroni test under the GLM procedure. The same procedure was applied to the overall analyses, including data from 2022–2024, to assess the effects of Year, Province, and their interaction (Year × Province) on parasitism rates. Mean separations were based on observed values rather than model estimates. Different lowercase letters within the same year indicate significant differences among provinces (*p* < 0.05), whereas different uppercase letters denote significant differences among overall yearly means (*p* < 0.05). To evaluate whether landscape context influenced parasitism in GSB, within-orchard and margin habitats were compared using the same statistical approach. For BMSB, habitat-based comparisons were not possible due to the very low number of naturally parasitized eggs, which resulted in an insufficient dataset for statistical evaluation.

### 2.4. Identification of Parasitoids

Following parasitoid emergence from egg masses of GSB and BMSB collected from the field, adult individuals were labeled based on their geographic locations (Table 1) and placed into sterile 2 mL cryotubes containing 70% ethanol. First, the parasitoid specimens were removed from ethanol, dried, and mounted on pinned cardboard. Then, the samples were examined under a Wild M5A (Wild Heerbrugg, Heerbrugg, Switzerland) stereo microscope, illuminated with LED spot lighting, and magnified up to 100×. For the identification of *Trissolcus* species, identification keys provided by Kozlov & Kononova [79], Talamas et al. [80], and Tortorici et al. [53] were used. The identification of *A. bifasciatus* was based on the keys in Askew & Nieves-Aldrey [81] and Peng et al. [82], while species belonging to the genus *Telenomus* were identified using Tortorici et al. [55]. Parasitoid species were identified and preserved by Dr. Francesco Tortorici at the University of Turin, Department of Agricultural, Forest and Food Sciences (Dipartimento di Scienze Agrarie, Forestali e Alimentari, DISAFA), Italy.

To verify the morphological identifications, nine representative specimens of the five most abundant species were subjected to DNA extraction. PCR reactions were performed using the Direct PCR Master Kit (Product Code: PCR-111S; Jena Bioscience GmbH, Jena, Germany) according to the manufacturer’s instructions to amplify the barcode region of the mitochondrial cytochrome c oxidase subunit I (COI) gene. The legs of the specimens were used directly for PCR amplification with the Direct PCR Master Kit. The PCR mixture contained 1 µL of specimen lysate, 2 µL of each primer at a concentration of 10 µM [LCO1490-puc (5′-TTTCAACWAATCATAAAGATATTGG-3′) and HCO2198-puc (5′-TAAACTTCWGGRTGWCCAAARAATCA-3′), Talamas et al. [83], 25 µL of Direct PCR Master Mix, and PCR-grade water to a final volume of 50 µL.

PCR conditions began with an initial denaturation at 94 °C for 5 min. This was followed by 5 cycles of denaturation at 94 °C for 30 s, annealing at 48 °C for 60 s, and extension at 72 °C for 60 s. Subsequently, 35 cycles were performed with denaturation at 94 °C for 30 s, annealing at 52 °C for 60 s, and extension at 72 °C for 60 s. A final extension step was carried out at 72 °C for 2 min. All PCR reactions were performed using a T100 thermal cycler (Bio-Rad, Hercules, CA, USA).

The resulting amplicons were purified and sequenced in both directions using the same primers by a commercial DNA sequencing company (Macrogen Inc., Seoul, Republic of Korea). The resulting sequences were read and edited using the MegAlign module of DNASTAR software (version 7.1.0; DNASTAR Inc., Madison, WI, USA). Consensus sequences were compared using the GenBank database (http://www.ncbi.nlm.nih.gov/genbank (accessed on: 20 September 2025)) and the BOLD Identification System (IDS; https://id.boldsystems.org/ (accessed on: 20 September 2025)) on the BOLD database. All sequences were submitted to the GenBank database.

## 3. Results

### 3.1. Egg Status and Parasitism Rates

**Figure 1:**
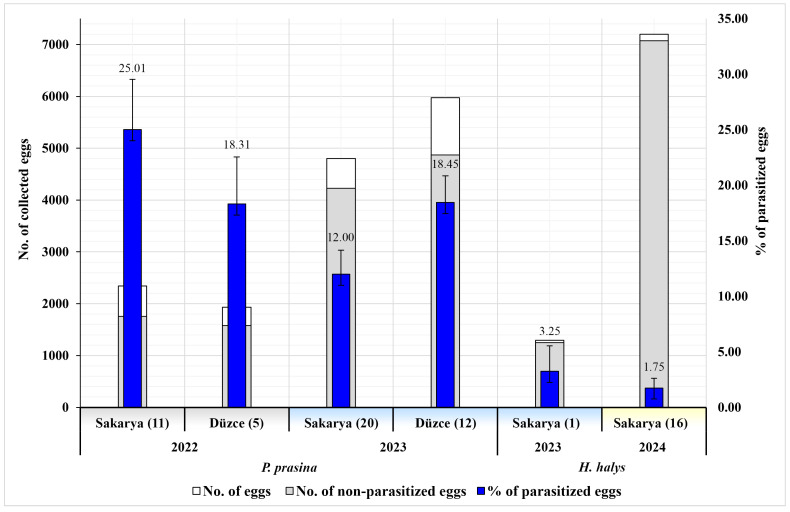
Distribution and parasitism status of GSB and BMSB egg numbers collected from hazelnut orchards in Sakarya and Düzce provinces during 2022–2024 (numbers in parentheses indicate the number of surveyed orchards).

To assess the egg status and year- and province-based variation in parasitism rates of GSB and BMSB, eggs were collected during a three-year survey across 65 hazelnut orchards in Sakarya and Düzce. GSB eggs were collected from 48 orchards and BMSB eggs from 17. A total of 23,541 eggs were obtained from both pests, of which 15,051 belonged to GSB (569 egg masses; mean ± SE number of eggs per mass: 26.45 ± 0.18) and 8490 to BMSB (308 egg masses; 27.56 ± 0.14 eggs per mass). The average parasitism rate of GSB eggs in 2022 and 2023 was 17.39% (2618 eggs), with 21.98% (940 eggs) in 2022 and 15.57% (1678 eggs) in 2023. For BMSB, the average parasitism rate in 2023 and 2024 was 1.98% (168 eggs), with 3.25% (42 eggs) in 2023 and 1.75% (126 eggs) in 2024. In Sakarya, 7144 GSB eggs were collected in 2022 and 2023, of which 16.27% (1162 eggs) were parasitized, while in Düzce, 18.41% (1456 eggs) of the 7907 collected eggs were parasitized. The overall parasitism rate of BMSB eggs in Sakarya was 1.98% (168 eggs). At the district level, the highest parasitism rate for GSB was recorded in Karasu (Sakarya) in 2022, with 33.96% (251 eggs), and in Arifiye (Sakarya) in 2023, with 60.42% (58 eggs) (Table 2). The GLM analysis revealed significant effects of Year (Wald χ^2^ = 18,439.84, *p* < 0.001), Province (Wald χ^2^ = 12,478.06, *p* < 0.001), and their interaction (Year × Province; Wald χ^2^ = 249.92, *p* < 0.001) on the parasitism rate of GSB. For BMSB, significant effects of Year (Wald χ^2^ = 12.28, *p* < 0.001) were detected. In addition, to assess whether landscape context influenced parasitism outcomes for GSB, parasitism rates were compared between within-orchard and margin habitats, and no statistically significant difference was detected between the two habitat types (Wald χ^2^ = 0.19, *p* = 0.661). Parasitism rates by years and provinces are presented in Figure 1.

### 3.2. Species Composition of Egg Parasitoids

**Figure 2:**
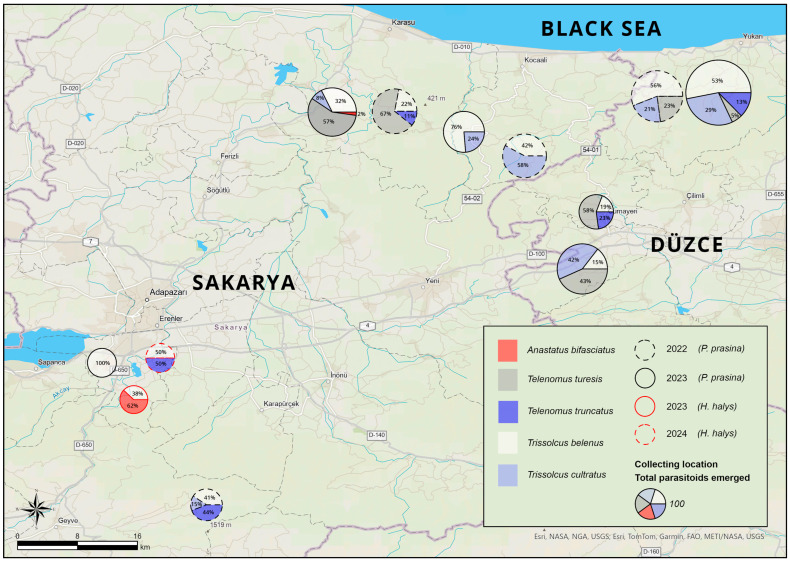
Proportional distribution of parasitoid species detected in egg masses of GSB and BMSB collected between 2022 and 2024 in Sakarya and Düzce provinces.

To determine the species composition and relative abundance of egg parasitoids associated with GSB and BMSB, the naturally laid eggs collected from hazelnut orchards were examined for parasitism. As a result of this examination, the eggs of GSB and BMSB were found to be parasitized by *T. belenus*, *T. cultratus*, *T. turesis*, *T. truncatus*, and *A. bifasciatus*. In GSB, the most dominant species across 2022 and 2023 was *T. belenus* (42.51%), followed by *T. turesis* (26.47%), *T. cultratus* (23.95%), *T. truncatus* (6.80%), and *A. bifasciatus* (0.27%). In 2022, the parasitism rates of GSB eggs were recorded as *T. belenus* (41.81%), *T. cultratus* (24.36%), *T. turesis* (26.60%), and *T. truncatus* (7.23%). In 2023, the corresponding rates were *T. belenus* (42.91%), *T. turesis* (26.40%), *T. cultratus* (23.72%), *T. truncatus* (6.56%), and *A. bifasciatus* (0.42%). As mentioned earlier, only a low parasitism rate (1.98%) was detected in BMSB eggs. Among the responsible species, *T. belenus* constituted the highest proportion (26.19%), followed by *T. truncatus* (16.67%) and *A. bifasciatus* (15.48%). In 2023, parasitism in BMSB eggs reached 38.10% by *T. belenus* and 61.90% by *A. bifasciatus*. In the following year, equal parasitism rates (22.22%) were observed for both *T. belenus* and *T. truncatus* (Table 2). The composition of egg parasitoids of GSB and BMSB, together with their spatial distribution across Sakarya and Düzce provinces and their temporal variations, is presented in Figure 2.

**Figure 3:**
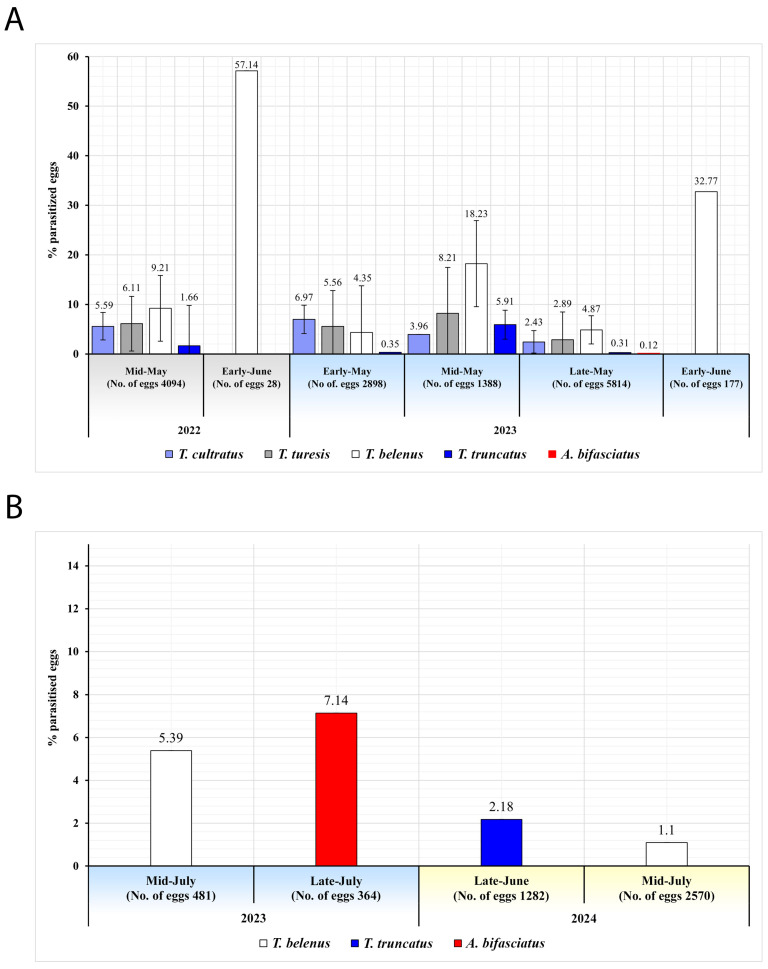
Yearly and monthly distribution of parasitoid species emerged from (**A**) GSB and (**B**) BMSB eggs in 2022–2024 (only months in which parasitoids were detected are included).

In mid-May 2022, the dominant parasitoid species emerging from 4094 GSB eggs was *T. belenus* (9.21%), followed by *T. turesis*, *T. cultratus*, and *T. truncatus*. In early May 2023, *T. cultratus* was the most prevalent (6.97%), while in mid-May, *T. belenus* again predominated (18.23%). By late May, *T. belenus* (4.87%) remained dominant, with other species recorded at lower frequencies, and in early June, it was the only species detected (32.77%). Across the entire survey, *T. belenus* showed the highest activity, highlighting its prevalence and potential role in suppressing GSB populations (Figure 3A). Parasitism of BMSB eggs in 2023–2024 remained low and restricted to three species. In 2023, *T. belenus* (5.39%) was detected in mid-July, and *A. bifasciatus* (7.14%) in late July. In 2024, parasitism was recorded only sporadically, with *T. truncatus* (2.18%) in late June, and *T. belenus* (1.10%) in mid-July. These results indicate that parasitism of BMSB eggs was both infrequent and taxonomically limited (Figure 3B).

### 3.3. Molecular Confirmation of Parasitoid Identification

To verify the morphological identification of the emerged parasitoid species, molecular analyses based on the COI gene were performed. BLAST+ (Version 2.17.0) analyses revealed 99–100% similarity with conspecific sequences available in both the GenBank and BOLD databases, thereby confirming the accuracy of the identifications. All consensus sequences obtained from representative specimens in this study were deposited into the GenBank database [PX241778 for *T. belenus* emerged from GSB; PX241779 for *T. belenus* emerged from BMSB; PX241782 and PX241783 for *T. cultratus* emerged from GSB; PX241776 and PX241777 for *T. turesis* emerged from GSB; PX241780 and PX241781 for *T. truncatus* emerged from GSB, and PX241784 for *A. bifasciatus* emerged from BMSB]. These molecular confirmations provide additional reliability for the species composition reported in the present study.

### 3.4. Discovery and Exploitation Efficiencies of Egg Parasitoids

**Figure 4:**
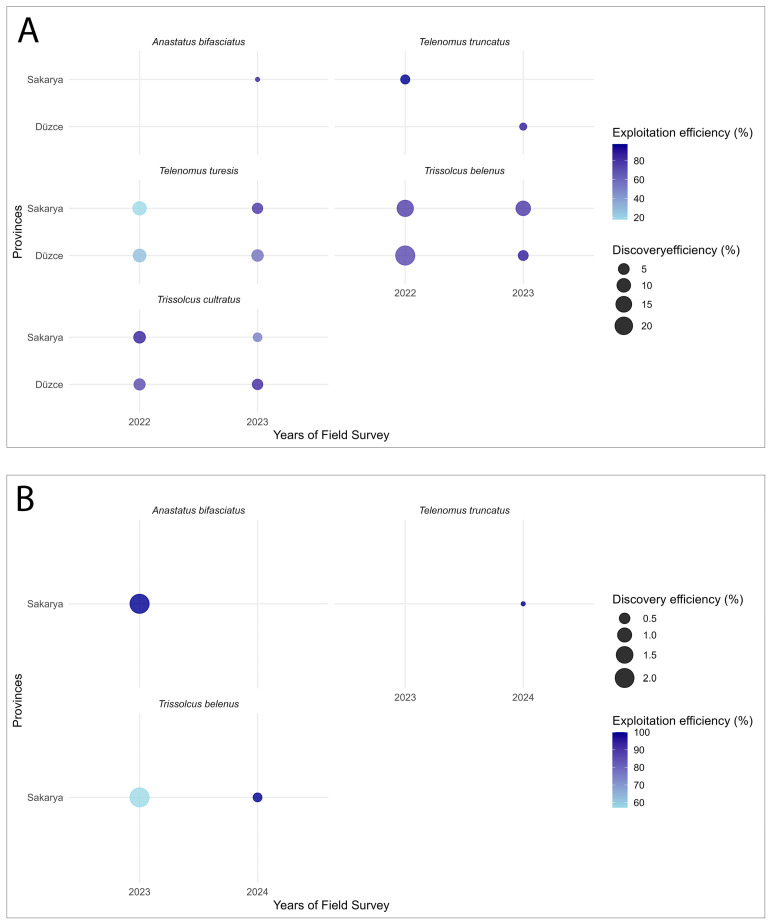
Comparative analysis of discovery and exploitation efficiencies of parasitoid species parasitizing (**A**) GSB and (**B**) BMSB egg masses across years and provinces.

To evaluate the host use performance of egg parasitoids, discovery and exploitation efficiencies were calculated for each species on GSB and BMSB eggs. In GSB eggs, *T. belenus* showed the highest exploitation (ratio of parasitized eggs to the total number of eggs in only those egg masses that were found to be parasitized; 89.83%) and discovery (ratio of the number of egg masses discovered by the parasitoid (i.e., those containing at least one parasitized egg) to the total number of collected egg masses; 24.88%) efficiencies, with significant differences in exploitation efficiency across years and Year × Province combinations (*p* < 0.05). However, the discovery efficiency of *T. belenus* did not vary significantly within the species across years and provinces (*p* > 0.05). It was followed by *T. cultratus* (88.10%; 6.35%) and *T. turesis* (79.97%; 9.62%), for which significant differences in exploitation efficiency were also detected across years, provinces, and Year × Province combinations (*p* < 0.05). Additionally, a significant difference in the discovery efficiency of *T. turesis* was also detected across years (*p* < 0.05). *Telenomus truncatus* (97.56%; 2.78%) and *A. bifasciatus* (87.50%; 0.08%) also displayed high exploitation efficiencies, although their discovery abilities were notably low and based on limited sample sizes (Figure 4A).

In BMSB eggs, relatively higher exploitation (up to 100%) and discovery (2.08%) efficiencies were observed for *A. bifasciatus* and *T. belenus*, while *T. truncatus* also showed a high exploitation rate (100%) but a very low discovery efficiency (0.14%). Because these results were derived from a limited number of egg masses, they should be interpreted with caution, and no statistical comparisons were possible. Overall, native parasitoid populations displayed substantially lower discovery efficiencies on BMSB eggs compared to GSB eggs (Table 3, Figure 4B).

## 4. Discussion

This study covers the identification of species parasitizing the naturally laid eggs of the native GSB and invasive BMSB in hazelnut orchards of Türkiye, particularly in Sakarya and Düzce provinces. Parasitoid species obtained over three years of surveys were identified, and their performance indices were evaluated. Additionally, our study provides the first record of *A. bifasciatus* parasitizing naturally laid GSB eggs, and of *T. truncatus* and *T. belenus* parasitizing BMSB eggs in Türkiye.

Two years of surveys, conducted in hazelnut and adjacent fruit orchards, revealed that the most dominant species from naturally laid GSB eggs was *T. belenus* (42.5%), followed by *T. turesis* and *T. cultratus* (both >20%). These identifications were robustly corroborated by molecular analysis—an approach recommended to enhance the reliability of species-level identifications in scelionid wasps [40,53]. The generated COI sequences showed 99–100% similarity with conspecific records in GenBank and BOLD, providing strong evidence for the accuracy of our findings and minimizing the likelihood of taxonomic error. In Northern Italy, including hazelnut orchards, *A. bifasciatus* was the most dominant species (40.84%) from naturally laid GSB eggs, followed by *T. belenus* (18.02%) and *T. cultratus* (9.16%) [50]. Similarly, Zapponi et al. [51] reported *A. bifasciatus* and *T. cultratus* equally dominant (28.95%) in naturally laid GSB eggs from Northern Italy and Switzerland, along with *T. turesis* (5.26%). In our study, *A. bifasciatus* was recorded from naturally laid GSB eggs, although only in Karasu (Sakarya) and at a low rate (0.42%). In the main hazelnut production areas of Türkiye, Ozdemir et al. [7] reported that frozen sentinel egg masses of GSB emerged *T. cultratus* (70.06%) as dominant, followed by *T. belenus* (15.75%), *T. turesis* (10.26%), *T. truncatus* (3.83%), and *Trissolcus* sp1 (0.01%). The species *T. cultratus*, *T. belenus*, and *T. turesis* were reported in our study, while *T. truncatus* was additionally detected in naturally laid GSB eggs (6.80%). This agrees with Tortorici et al. [55], who identified *Telenomus* sp1 in GenBank (OK562072) as *T. truncatus*. To our knowledge, this is the first field record of this species parasitizing GSB eggs in Türkiye. The widespread presence of *T. belenus* was also confirmed in all hazelnut areas, consistent with Kurt [54] and Moraglio et al. [50]. In Düzce, Atak et al. [84] reported *T. turesis* and *T. belenus* on *Eurygaster integriceps* Puton (Hemiptera: Scutelleridae) eggs, and our findings confirmed these parasitoids across all surveyed areas. Unlike our results, fresh sentinel GSB egg masses in Italy were parasitized by *T. semistriatus* and *T. turesis* at equal rates (50%) [40]. In addition, *T. kozlovi* has been reported in naturally laid GSB eggs in Russia and Northern Italy [40,85]. The hyperparasitoid *Acroclisoides sinicus* (Huang & Liao) (Hymenoptera: Pteromalidae), associated with *Trissolcus* spp., has also been reported from naturally laid GSB eggs [50,51], often linked to *T. mitsukurii,* but showing low efficiency on *T. japonicus* under laboratory conditions [86]. It was not found in our study. Moreover, in BMSB eggs, *T. cultratus* occasionally behaves as a facultative hyperparasitoid, successfully developing under interspecific competition [65,87]. While this complicates estimates, it does not compromise overall parasitization success. Further studies are needed to clarify its role in GSB eggs. In addition, *T. japonicus* and *T. mitsukurii*, the adventive species in the classical control of BMSB, were rarely reported to parasitize naturally laid GSB eggs [51]. In Türkiye, Ozdemir et al. [7] recorded 9.30% parasitism in 10,386 frozen sentinel GSB eggs. By contrast, our study, based on 15,051 naturally laid eggs, recorded a 17.39% rate. Although species composition partly overlapped, *A. bifasciatus* was additionally found in naturally laid eggs, and parasitism rates were higher. Naturally laid eggs provide host–plant semiochemical cues that facilitate parasitoid detection [45,46,88,89], whereas sentinel eggs, produced and stored without plant association, lack these cues and are less easily parasitized [70,71,74,90,91]. Other studies also found higher rates in naturally laid eggs: Haye et al. [47] reported 22.4% parasitism from 232 fresh sentinel eggs, Zapponi et al. [51] 51.35% from 74 naturally laid eggs, and Moraglio et al. [50] 46.55% from 4760 naturally laid eggs. Despite collecting more eggs, our study yielded a lower rate (17.39%), likely due to heavy insecticide use. Intensive treatments against GSB, BMSB, and hazelnut weevil (*Curculio nucum* L., Coleoptera: Curculionidae) from May onwards coincide with oviposition and suppress parasitoids. Recent BMSB outbreaks increased broad-spectrum insecticide use, further reducing performance [41]. In addition, our analysis showed that landscape context did not influence parasitism in GSB, with similar rates in within-orchard and margin habitats. A comparable pattern was reported by Zapponi et al. [92] for BMSB, where habitat context also did not significantly affect parasitism of naturally laid eggs.

Numerous studies have identified parasitoids of BMSB eggs [40,47,48,50,51,68,93,94]. The low parasitism rate (1.98%) in our study matches patterns from recently invaded areas. In Northern Italy, sentinel (fresh) BMSB eggs showed 1–3% parasitism (11,841 eggs), all by *A. bifasciatus* [49]. Natural BMSB eggs later showed parasitism rising from ~12% in 2016–2017 to 21% in 2018, with *A. bifasciatus* dominant each year [40]. The increase coincided with the first detection of *T. japonicus* in 2018, when parasitism reached 34% at one site. Similarly, Zapponi et al. [51] reported 22.40% parasitism from 4348 naturally laid BMSB eggs, with *A. bifasciatus* most common (64.06%). Haye et al. [47] also identified *A. bifasciatus* as the only native species with high performance (34.1%) on fresh eggs, while *T. cultratus* and *T. semistriatus* achieved only 2.27% development in frozen eggs. In our study, *A. bifasciatus* was also recorded parasitizing BMSB eggs, consistent with reports from cherry laurel (*Prunus laurocerasus*) and vineyard in Türkiye [57]. Exotic species were also detected: *T. mitsukurii* (33.36%) and *T. japonicus* (15.61%) on BMSB eggs [51]. Although adventively established *T. japonicus* and *T. mitsukurii* in Europe have been reported to parasitize eggs of native species only rarely, determining the official host ranges of exotic parasitoids before their intentional release, especially those not yet established in the field, is critical for assessing potential non-target effects [95]. The tendency of adventive parasitoids to attack native hosts may weaken their pressure on the invasive pest and thereby reduce biological control efficacy [96]. Moreover, host shifting can generate a trade-off, such as poor performance on the novel host and fitness loss upon returning to the ancestral host; this may lead to changes in host preference and ultimately influence control success [96,97,98,99,100,101,102,103]. Therefore, the release of exotic species for classical biological control should be carefully evaluated by taking their potential ecological risks into account. Over the past decade, however, extensive laboratory and field studies have identified *T. japonicus* as the most promising classical biological control agent of BMSB, owing to its high parasitism rates and close co-evolutionary association with this host. Seasonal parasitism, host-specificity, and fundamental host-range tests conducted in Asia, Europe, and North America indicate that *T. japonicus* is strongly biased toward BMSB, and that non-target effects, while possible under worst-case laboratory conditions, are likely to remain within acceptable biosafety thresholds when releases are carefully regulated and monitored [104,105,106,107]. In 2023, the Turkish Ministry of Agriculture began mass releases of *T. japonicus* in the Eastern Black Sea region, and in 2024, releases were expanded to include our study areas, and by 2025, a total of 1,156,000 parasitoids had been released [75]. However, no natural parasitism of BMSB eggs by *T. japonicus* has yet been reported in Türkiye. During the first outbreak, Ozdemir [56] suspended 600 BMSB eggs on host plants under cages and found only 1.73% parasitism, attributed to *T. turesis*.

Considering all results together, the findings reveal clear host-linked specificity among the native parasitoids detected. Consistent with its dominance and strong performance indices, *T. belenus* appears highly adapted to the native host, GSB. Likewise, *T. turesis* reliably parasitized GSB eggs across the study area, indicating stable associations shaped by long co-evolutionary history [51,91]. From an applied perspective, conserving and augmenting these native parasitoid communities—through careful insecticide timing and the maintenance of vegetative refugia—may enhance natural regulation of GSB populations in hazelnut orchards [51,108]. In contrast, parasitism on BMSB remained very low and taxonomically limited, aligning with global evidence that native European and Anatolian parasitoids show poor compatibility with this invasive species [51,91]. Our findings indicate that although species such as *T. truncatus*, *T. belenus* and *A. bifasciatus* parasitized BMSB eggs, their lack of host-linked specificity resulted in limited development on this host and produced very few successful progeny. This pattern suggests that invasive species may represent an evolutionary trap for native parasitoids [67,73,109,110,111]. In such traps, invasive hosts are accepted but do not support development, causing energy and time loss [67,112]. Thus, the unsuitability of native parasitoids for BMSB not only reduces control effectiveness but may also decrease their pressure on natural hosts such as GSB, creating indirect effects. Consequently, BMSB may disrupt native parasitoid–host dynamics and trigger complex biotic interactions, potentially leading to increased native pest populations [67,113,114].

## 5. Conclusions

This study investigated natural parasitism and species composition of egg parasitoids on GSB and the invasive BMSB in hazelnut orchards and neighboring fruit orchards in Sakarya and Düzce, Türkiye’s main hazelnut production areas. Over three years, five parasitoid species were identified. Notably, *A. bifasciatus* was recorded for the first time from GSB, and *T. belenus* and *T. truncatus* from BMSB in Türkiye. Native parasitoids, particularly *T. belenus* (42.51%), *T. turesis* (26.47%), and *T. cultratus* (23.95%), showed high dominance and increased parasitism rates compared to previous studies, highlighting their potential as biological control agents of GSB. In contrast, parasitism in naturally laid BMSB eggs was low (1.98%), indicating limited effectiveness of native species against this invasive pest. Although *T. belenus* and *T. truncatus* parasitized BMSB eggs, low progeny emergence suggests BMSB might act as an evolutionary trap. *A. bifasciatus*, a generalist species, was detected on both hosts but at low frequency. *T. japonicus* was not recovered, implying it has not yet been established. Although native parasitoids are generally considered ineffective against BMSB due to evolutionary mismatch, their natural parasitism on GSB eggs is promising for long-term management. The findings clarify parasitoid composition and natural parasitism patterns, demonstrating that conserving and augmenting native parasitoids can contribute to more sustainable management for GSB and reduced insecticide dependence in Turkish hazelnut orchards.

## Figures and Tables

**Table 1:** Fruit orchards in Türkiye, where parasitoids of GSB and BMSB were detected in 2022–2024.

Year	Province	District (No. of Location)	Host	Latitude	Longitude
2022	Sakarya	Kocaali (4)	*Corylus avellana*	40°56′50.3″ N	30°51′13.3″ E
				40°56′48.3″ N	30°51′22.0″ E
				40°56′52.3″ N	30°51′21.9″ E
				40°57′8.5″ N	30°51′16.9″ E
		Karasu (4)		41°0′2.9″ N	30°41′57.9″ E
				41°0′3.2″ N	30°41′59.0″ E
				41°0′5.2″ N	30°41′58.7″ E
				41°0′1.9″ N	30°41′57.7″ E
		Geyve (1)		40°31′44.2″ N	30°28′20.8″ E
	Düzce	Akçakoca (5)		41°1′7.4″ N	31°0′45.2″ E
				41°1′9.5″ N	31°0′45.7″ E
				41°1′10.2″ N	31°0′42.4″ E
				41°1′10.9″ N	31°0′50.3″ E
				41°1′9.6″ N	31°0′50.9″ E
2023	Sakarya	Kocaali (5)	*C. avellana*	40°58′33.9″ N	30°46′51.8″ E
			*Prunus spinosa*	40°58′38.8″ N	30°46′51.2″ E
			*C. avellana*	40°56′50.3″ N	30°51′13.3″ E
				40°56′48.3″ N	30°51′22.0″ E
				40°57′08.5″ N	30°51′16.9″ E
		Karasu (4)		41°00′03.0″ N	30°41′57.9″ E
				40°59′59.4″ N	30°41′53.3″ E
				40°59′29.1″ N	30°41′09.3″ E
				40°59′29.6″ N	30°40′44.0″ E
		Arifiye (2)		40°41′56.8″ N	30°20′49.6″ E
				40°42′03.1″ N	30°20′44.0″ E
	Düzce	Akçakoca (2)		41°01′07.4″ N	31°00′45.2″ E
				41°01′09.5″ N	31°00′45.7″ E
		Dereköy (5)		40°48′41.8″ N	30°55′22.6″ E
				40°48′38.1″ N	30°55′40.9″ E
			*C. avellana* and *P. avium*	40°48′38.0″ N	30°55′39.9″ E
			*C. avellana*	40°48′41.8″ N	30°55′42.4″ E
				40°48′29.4″ N	30°55′39.6″ E
		Cumayeri (1)		40°52′48.4″ N	30°56′22.6″ E
2024	Sakarya	Arifiye (2)	*C. avellana*	40°41′57.2″ N	30°20′49.6″ E
			*Actinidia deliciosa*	40°42′17.9″ N	30°25′01.1″ E

**Table 2:** Summary of naturally laid egg masses and eggs of GSB and BMSB collected from fruit orchards in Türkiye (2022–2024), showing the proportions of parasitized, hatched, unhatched, sucked, and broken eggs, and the species composition of emerging egg parasitoids.

Year	Pest Species	Province	District (No. of Orchards)	Period	No. Egg Masses	No. Eggs	% Hatched	Eggs Parasitized % (No. of Parasitoids)	% Sucked	% Broken	% Unhatched	Species Composition of Parasitoids (%)				
												*Trissolcus belenus*	*Trissolcus cultratus*	*Telenomus turesis*	*Telenomus truncatus*	*Anastatus bifasciatus*
**2022**	*P. prasina*	Sakarya	Kocaali (5)	13–20 May	48	1301	54.42	18.60 ± 5.54 b (242)	8.15	0.77	0.54	42.15	57.85	-	-	-
			Karasu (4)		27	739	34.64	33.96 ± 8.60 a (251)	2.3	0.27	24.49	21.91	-	67.33	10.76	-
			Geyve (2)		12	303	45.87	30.69 ± 14.21 ab (93)	8.58	0	1.65	40.86	15.05	-	44.09	-
		Düzce	Akçakoca (5)	18 May–10 June	71	1933	65.65	18.31 ± 4.24 c (354)	6.57	1.76	3.88	55.93	21.19	22.88	-	-
	**Total**				**158**	**4276**	**55.47**	**21.98 ± 3.13 A (940)**	**6.45**	**1.08**	**6.27**	**41.81**	**24.36**	**26.60**	**7.23**	**-**
**2023**	*P. prasina*	Sakarya	Kocaali (8)	3–26 May	83	2112	82.58	9.61 ± 3.21 e (203)	2.56	2.98	0.14	76.35	23.65	-	-	-
			Karasu (10)	2–31 May	99	2512	37.06	12.54 ± 3.07 d (315)	0.04	2.67	0	32.38	8.25	57.14	-	2.22
			Arifiye (1)	10–30 June	5	96	33.33	60.42 ± 9.99 a (58)	0	16.67	0	100	-	-	-	-
			Geyve (1)		3	81	100	0 (0)	0	0	0	-	-	-	-	-
		Düzce	Akçakoca (5)	8–29 May	108	2931	71.95	21.08 ± 3.61 c (618)	0	1.84	0	53.07	28.96	5.34	12.62	-
			Gümüşova (6)	3 May–11 June	95	2550	81.69	13.49 ± 3.35 d (344)	0	1.14	0	14.53	42.15	43.31	-	-
			Cumayeri (1)	15–30 May	18	493	37.32	28.40 ± 10.29 b (140)	0	6.09	0	19.29	-	57.86	22.86	-
	**Total**				**411**	**10** **,775**	**67.32**	**15.57 ± 1.63 B (1678)**	**0.51**	**2.40**	**0.03**	**42.91**	**23.72**	**26.40**	**6.56**	**0.42**
	**Totals for *P. prasina* in 2022 and 2023**				**569**	**15** **,051**	**63.96**	**17.39 ± 1.47 (2618)**	**2.20**	**2.03**	**1.80**	**42.51**	**23.95**	**26.47**	**6.80**	**0.27**
	*H. halys*	Sakarya	Arifiye (1)	20 June–30 July	50	1293	95.82	3.25 ± 2.28 A (42)	0	0	0	38.1	-	-	-	61.9
**2024**	*H. halys*	Sakarya	Arifiye (16)	25 June–16 July	258	7197	96.3	1.75 ± 0.86 B (126)	0	0	0	22.22	-	-	22,22	-
**Totals for *H. halys* in 2023 and 2024**					**308**	**8490**	**96.23**	**1.98 ± 0.81 (168)**	**0**	**0**	**0**	**26.19**	**-**	**-**	**16.67**	**15.48**

Different lowercase letters within the same year indicate significant differences among provinces, according to the Bonferroni test (*p* < 0.05). Different uppercase letters indicate significant differences between the overall means of years based on the GLM (binomial, logit link).

**Table 3:** Average (±standard error) discovery and exploitation efficiencies of egg parasitoids on naturally laid egg masses of GSB and BMSB across all sampled hazelnut orchards during 2022–2024.

Parasitoid Species	Pest	Year	Province	Discovery Efficiency (%)	GLM (Discovery Efficiency, Wald χ^2^, *p*)	Exploitation Efficiency (%)	GLM (Exploitation Efficiency, Wald χ^2^, *p*)
*T. belenus*	*H. halys*	2023	Sakarya	2.08 ± 0	N/A *	57.14 ± 0	N/A
		2024		0.33 ± 0.33		100 ± 0	
	*P. prasina*	2022	Düzce	24.88 ± 15.53 a	Year χ^2^ = 0.55 (*p* = 0.459); Province χ^2^ = 0.35 (*p* = 0.553); Year × Province χ^2^ = 0.44 (*p* = 0.507)	73.51 ± 8.11 b	Year χ^2^ = 3.97 (*p* = 0.046); Province χ^2^ = 1.20 (*p* = 0.273); Year × Province χ^2^ = 18.28 (*p* < 0.001)
			Sakarya	16.46 ± 9.55 a		77.34 ± 11.10 ab	
		2023	Düzce	3.75 ± 1.77 a		89.83 ± 2.97 a	
			Sakarya	12.02 ± 6.12 a		79.92 ± 5.70 b	
*T. cultratus*	*P. prasina*	2022	Düzce	5.57 ± 2.19 a	Year χ^2^ = 0.004 (*p* = 0.948); Province χ^2^ = 0.86 (*p* = 0.354); Year × Province χ^2^ = 1.14 (*p* = 0.285)	72.12 ± 15.04 b	Year χ^2^ = 34.19 (*p* < 0.001); Province χ^2^ = 5.49 (*p* = 0.019); Year × Province χ^2^ = 230.68 (*p* < 0.001)
			Sakarya	6.35 ± 2.90 a		88.10 ± 9.95 a	
		2023	Düzce	4.51 ± 1.77 a		84.46 ± 5.86 a	
			Sakarya	2.38 ± 1.66 a		51.97 ± 20.25 c	
*T. turesis*	*P. prasina*	2022	Düzce	8.25 ± 4.68 a	Year χ^2^ = 5.81 (*p* = 0.016); Province χ^2^ = 1.50 (*p* = 0.221); Year × Province χ^2^ = 2.51 (*p* = 0.113).	28.18 ± 12.87 c	Year χ^2^ = 116.22 (*p* < 0.001); Province χ^2^ = 7.12 (*p* = 0.008); Year × Province χ^2^ = 4.88 (*p* = 0.027)
			Sakarya	9.62 ± 5.19 a		17.83 ± 7.31 c	
		2023	Düzce	6.12 ± 3.04 a		57.57 ± 16.24 b	
			Sakarya	4.39 ± 2.95 a		79.97 ± 9.31 a	
*T. truncatus*	*H. halys*	2024	Sakarya	0.14 ± 0.14	N/A	100 ± 0	N/A
	*P. prasina*	2022	Sakarya	2.78 ± 1.90 a		97.56 ± 2.44 a	
		2023	Düzce	1.02 ± 0.69 a		89.00 ± 11.00 b	
*A. bifasciatus*	*H. halys*	2023	Sakarya	2.08 ± 0	N/A	100 ± 0	N/A
	*P. prasina*			0.08 ± 0.08		87.50 ± 0	

In each column, values followed by the same letter are not significantly different (Bonferroni test, *p* < 0.05), under a GLM procedure with binomial distribution and logit link. * The model estimation could not be computed due to numerical problems.

## Data Availability

The datasets generated during and/or analyzed during the current study are available from the corresponding author upon reasonable request.
